# Key features of blend denim fabrics performance with dyed weft silk yarn and different weave structures

**DOI:** 10.1038/s41598-024-61944-0

**Published:** 2024-05-25

**Authors:** Yehya Youssef, Abdalla Mousa, Hany Kafafy, Tamer Hamouda, Samiha Abo El-Ola

**Affiliations:** 1 Dyeing, Printing and Textile Auxiliaries Department, Textile Research and Technology Institute, National Research Centre, 33 El-Behouth St, Dokki, P.O. 12622, Giza, Egypt; 2 Textile Research and Technology Institute, National Research Centre, 33 El-Behouth St, Dokki, P.O. 12622, Giza, Egypt; 3 Wilson College of Textiles, NC State University, Raleigh, NC 27695 USA; 4 Protein and Man-Made Fibers Department, Textile Research and Technology Institute, National Research Centre, 33 El-Behouth St, Dokki, P.O. 12622, Giza, Egypt

**Keywords:** Chemistry, Engineering, Materials science

## Abstract

This research aimed to weave the warp indigo-dyed cotton yarn with un-dyed or dyed silk yarn and analyze the impact of different weft yarn structures on the properties of denim fabrics. The dyed silk yarn was performed by a selection of different anionic indigo and non-indigo blue dyestuffs. The dyeing shades of the anionic Indigo Carmine dye on silk exhibited high build-up at the acidic pH range 2–2.5 with poor washing fastness and even so, the cationic aftertreatment of the dyed silk samples showed un-matched color with indigo-dyed cotton yarns. The dyeing properties of two commercial non-indigo reactive and acid dyes on silk add other advantages. To ascertain the dyeing shades evaluation of the non-indigo dyes on silk, two sets of blended denim fabrics were investigated. The first set included a weft-wise silk yarn dyed with reactive dye **RB 5**, and the second set included silk yarns dyed with acid dye **AB 193**. Weaving of the blended fabrics was carried out in Twill 3/1, 3/2 Z,and Satin 5_3_ patterns and exhibited significant color effects of the dyed silk yarns to those of the un-dyed control samples. The dyeing shades of the non-indigo **RB 5** and **AB 193** dyed weft-wise silk yarns were found to be matched in color performance with the conventionally indigo- dyed cotton yarns. Ultraviolet resistance of the blend denim fabrics was evaluated, showing significant improvement in UPF of the weft-wise dyed silk. The study claimed that the dyed silk yarns a good candidate for newly developed blend denim fabrics.

## Introduction

The global cotton denim market is challenging, forcing the industry to use and develop sustainable natural fibers that are environmentally friendly, comfortable, and fashionable^1–9^. Denim fabrics are traditionally produced from 100% cotton with indigo-dyed warp yarn and unbleached or undyed weft yarn for jeans, work clothes, and casual wear. Customers of all ages prefer denim garments because of their unique properties like high moisture absorptivity, effortless wearability, and breathability^10–15^.

Increased environmental considerations and awareness of sustainability inspired the need for innovative denim products with comfort, fashionable performance, and functional finishing, closely related to thermal and electrical conductivity, antimicrobial properties, and UV protection^16–18^. Also, there is a need for comfort with a soft touch and a lightweight as well as stretching fabric. In this context, comfort properties are one of the vital concerns for fabric quality, which largely depends on the fiber compositions and structural characteristics of denim fabrics^19–21^. Therefore, proper and developed denim fabric constructions have been designed to achieve both fashion and comfort performance-driven. It is also known that fibre type, yarn properties, fabric structure, finishing treatments, apparel designs, and clothing conditions are key factors contributing to the comfortability of denim products^22–29^.

It is known that the use of natural fibers in denim garments is drawing attention because of their outstanding performance. Understanding fabric properties that influence properties is essential for designing denim fabrics that provide comfort to the wearer^26–29^. Denim products are primarily composed of twilled weave fabrics, with weft yarns floating across the fabric surface's warp direction of the fabric surface. These floating weft yarns help to impart better fabric properties which predominantly influence the heat exchange between skin and the fabric. So, the comfort evaluation of the weft-wise direction of different natural fabric types could be considered a key factor for fiber selection.

Silk fibers have excellent properties such as softness, strength, lightweight, moisture absorption, quick drying, breathability, and good thermal and electrical performance. Because of its hygroscopicity, anti-ultraviolet, biodegradability, and biocompatibility with the human body, silk, and other natural fibers were proposed to be used in denim products^30,31^.

Analyzing these types of blended denim products might enable us to understand their suitability for use and the potential advantages for their application in textile industries. However, the number of research activities focusing on denim fabrics produced from silk fiber is relatively low. On the other hand, the overall moisture management capacity indexes of silk fabric are found to range from “very good” to “excellent” category, indicating the suitability of silk yarn to skin fit and active-wear applications. This article aimed to investigate the denim fabric properties comprising a blended silk weft yarn with the typical warp cotton indigo-dyed yarn. Applications of undyed or dyed types of silk weft yarns were primarily studied. The performance of conventional indigo-dyed warp cotton yarns combined with different shades of anionic indigo and non-indigo blue dyed silk yarns was investigated. For this purpose, the typical warp cotton-indigo-dyed yarns blended with undyed and dyed types of silk weft yarns were fabricated and examined in different structural compositions. The color effects of the proposed bended denim fabrics patterns claimed as good candidate materials for newly developed denim products.

## Experimental details

### Materials and methods

A 100% ring-spun carded cotton yarn of yarn count 16^s^/1 Ne used in the production of all samples.

Control blend fabrics of weftwise un-dyed silk yarns and typical indigo-dyed warp cotton yarns fabrics were produced in 3/1 Z Twill (**DF1**), 3/2 Z Twill (**DF2)** and Satin 5_3_ **(DF3)** weave structure patterns, the fabric was produced with 28 ends/cm and 38 picks/cm for cotton. The average basis weigh of the produced fabrics are 190 g/cm. Table 1 depicts the weave structures of the control blend denim fabrics composed of indigo-dyed warp cotton yarn and un-dyed weft silk yarn.

**Table 1:** Weave structures of the control blend denim fabrics composed of indigo-dyed warp cotton yarn and un-dyed weft silk yarn.

Sample code	Weave setting	Weave structure
**DF1**	Twill 3/1 Z	
**DF2**	Twill 3/2 Z	
**DF3**	Satin 5_3_	

Cotton yarn was dyed with Indigo dye (DenimBlu^30^) by the rope dyeing method described by Bluconnection, Singapore, as mentioned below. Weft yarns of undyed and dyed silk (70/2 Ne) were used. Indigo Carmine dye (C.I. Acid Blue 74), purchased from Sigma Aldrich, one commercial reactive dye with the trade name Remazol Black B, C.I. Reactive Black 5, **RB 5)**, comprising a homo-bifunctional Bis-(VS) reactive system, supplied by Ohyoung Industrial Co., Ltd., South Korea and one commercial acid dye (Ryian Blue T2R, C.I. Acid Blue 193, **AB 193**), supplied by Synthesia, CZ, were used as received. The chemical structures of these dyes are illustrated in Table 2.

**Table 2:** Commercial names and chemical structures of the indigo and non-indigo dyes.

CI Generic name	Commercial name	Dye structure
C.I. Vat Blue 1 (Indigo Dye)	IndigoBlu30	
C.I. Acid Blue 74 (Indigo Carmine)	Indigo Carmine	
C.I. Acid Blue 193 (**AB 193**)	Ryian Blue T2R (Synthesia)	
C.I. Reactive Black 5 (**RB 5**)	Remazol Black B (DyStar)	

### Dyeing procedure

#### Indigo dyeing process of warp cotton yarn

Dyeing of warp cotton yarns with Indigo dye was conducted in four stages as following:^2,32^

**Table:** 

a) Pretreatment stage	b) Dye bath preparation at 70 °C
25 g/L NaOH 50% 3.0 g/L wetting agent 2.0 g/L chelating agent for 15 min at 60 to 80 °C , followed by hot and cold rinse	40 g/L Glauber’s or common salt 20.0 g/L NaOH 50% 3.0 g/L Sodium hydrosulfite 2–5 g/L DenimBlu (added as stock indigo dye) 1.0 g/L wetting agent 2.0 g/L dispersing agent, liquid form 2.0 g/L chelating agent
c) Dyeing Stage	d) Rinsing/Peroxide Oxidation
50° C dyeing temperature 30 s. dipping time (4 dips) dyeing pH 12.0 at 50 °C 2–4 min Air-oxidation	1 time rinse at room temperature with overflow 1 time oxidation at 60 °C with 1.0 g/L hydrogen peroxide 50%, 1.0 g/L acetic acid 60% (pH 5.5 to 6.0). 2 time rinse at 60 °C with counter flow Air-dried

**Figure 1:**
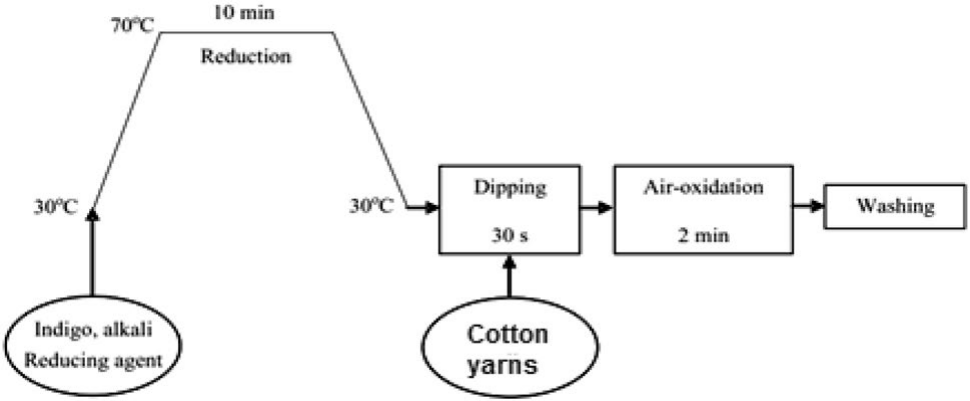
Schematic indigo dyeing of cotton yarn.

The reduction temperature was set at 70 °C for 10 min. After that the dyebath was cooled down to 40 °C and the dyeing was consequently done by successive dipping warp yarns through several troughs of indigo dye liquors for 20–30 s, then air oxidized for 2–4 min, as shown in Fig. 1.

### Dyeing of weft silk yarns

Silk yarn was initially subjected to washing in a cone dyeing machine at a liquor ratio 20:1 using 1g/l nonionic detergent and 2g/l sodium bicarbonate for 30 min at 60 °C, then neutralized with diluted acetic acid solution (1 ml/l) and air dried.

#### Dyeing silk yarn with Indigo Carmine dye

Dyeing of silk yarn using Indigo Carmine was carried out at a liquor ratio of 40:1. The dyebath was adjusted at different pH 2, 2.5, 3, 4 and 6 with different dye concentrations 1–5% owf. Dyeing was started at 30 °C for 15 min. The temperature was then raised to 90 °C, over 15 min and continued for further 30 min. After rinsing, the dyed samples were subjected to cationic after-treatment to fix the dye, then soaped in a an aqueous solution containing 2 g/l nonionic detergent for 30 min at liquor ratio 50:1, rinsed and air- dried.

#### Dyeing silk yarn with non-indigo dyes

##### Dyeing with reactive dye

Silk yarn samples were dyed with Remazol Black B, C.I. Reactive Black 5 (**RB 5**) (DyStar, Egypt) which was carried out at neutral pH 7 and at a liquor ratio 40:1. The initial dyebath was set at 40 °C for 10 min. The temperature was raised to 90 °C in 30 min. During this period 60 g/l sodium sulphate was added in two portions at an interval of 15 min. Dyeing was continued at these conditions for a further 90 min. After dyeing all the dyed samples were rinsed with water and air dried.

##### Dyeing with acid dye

**Figure 2:**
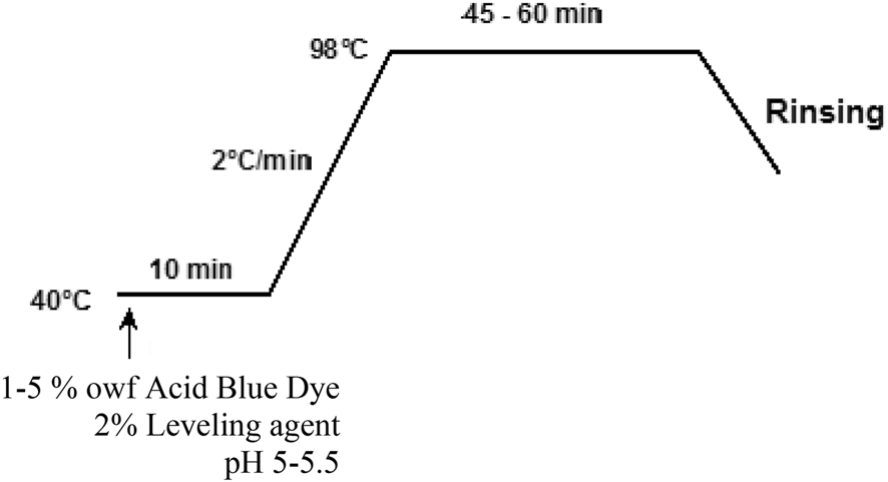
Dyeing of silk yarn with **AB 193.**

Silk yarn samples were dyed with the commercial metal complex acid dye **AB 193** at 1–5% owf dye concentration and at a liquor ratio of 20:1; the dyeing method used is exhibited in Fig. 2. At the end of dyeing, the dyed samples were removed, rinsed thoroughly washed in water and air dried.

### Blend denim fabric preparations

Two sets **A** and **B** of blend denim fabrics of weft-wise dyed silk yarns and typical indigo-dyed warp cotton yarns based fabrics were produced in 3/1 and 3/2 Z Twill and Satin weave structures. Fabrics in set **A** included silk yarns that were subjected to dyeing with the reactive dye (**RB 5**), at 4% owf shade, while set **B** included silk yarns dyed with the acid dye (**AB 193**), at 4% owf shade. The production parameters concerning the weaving process of the categorized samples of set **A** (**DF1A**, **DF2A** and **DF3A**) and samples of set **B** (**DF1B**, **DF2B** and **DF3B**) were identical to those of the control fabrics **DF1**, **DF2** and **DF3**, respectively, as presented in Table 1.

### Measurements and testing

#### Color strength and CIELAB

The relative color strength (K/S) of the dyed samples were measured using a Hunter Lab Ultra Scan PRO spectrophotometer (USA) under illuminant D65, 10° standard observer. The K/S values were measured by the light reflectance technique using Kubelka–Munk Eq. (1).

1 $${\text{K}}/{\text{S}}= \frac{{(1-{\text{R}})}^{2}}{2{\text{R}}}$$

where,

K = Absorption coefficient, and S = Scattering coefficient.

R = Decimal fraction of the reflection of the dyed fabric.

The color readings of all dyeings were expressed in the CIELAB color space system (often denoted as L*, a*, b*, C*and *h* coordinates). In which, L* represents lightness or darkness of the sample (a higher lightness value represents a lower colour yield); a* denotes redness if positive value or greenness if negative; b*represents yellowness if positive or blueness if negative.

#### UV protection

The UV protection factor (UPF) was determined on the fabricated blend denim samples (5 × 5 cm). Each sample was fixed on a common slide frame and placed in a Jasco UV/VIS Spectrophotometer V-560 equipped with an integrating sphere to measure both direct and diffuse transmitted light. Each sample was positioned at right angles to the light beams. Transmission measurements were made in the 290–400 nm range with a 1 nm step. UPF was calculated according to Eq. (2).

2 $$UPF= \frac{{\sum }_{290}^{400}{E}_{\lambda }{S}_{\lambda }{\Delta }_{\lambda }}{{\sum }_{290}^{400}{E}_{\lambda }{S}_{\lambda }{T}_{\lambda }{\Delta }_{\lambda }}$$

where S_λ_ is the solar spectral irradiance for a typical summer’s day, E_λ_ is the CIE erythemal spectral effectiveness, T_λ_ is the spectral transmittance of each fabric and Δ_λ_ is the wavelength step in nm.

The values of protection factors are in range 15–50 where the higher number represents a better protection (Table 3)^33–35^.

**Table 3:** UPF ratings and protection categories according to the Australian/New Zealand standard.

UPF range	Protection category
< 15	Insufficient
15–24	Good
25–39	Very good
40–50, 50 +	Excellent

#### Fastness testing

The color fastness of the produced denim fabrics, after washing-off using 2 g/l nonionic detergent at 80 °C for 15 min, were tested in accordance with ISO standard methods^36–38^. The wash fastness test was assessed in accordance with the standard method ISO 105-C06 B2S (4g/l of ECE detergent, 1 g/1 of sodium perborate, 25 steel balls) at 50 °C for 30 min and at a liquor ratio of 50:1. Fastness to acidic and alkaline perspiration was determined with a perspirometer set at specific pressure, temperature and time in accordance with ISO 105-E04. Any change in color (*CC*) of the specimens and color staining of the adjacent cotton (*SC*) and wool (*SW*) fiber was then assessed with the corresponding ISO grey scales for color change and staining. Light fastness was also assessed using a Xenon arc lamp test in accordance with ISO 105-B02.

## Results and discussion

Typical denim fabric is mostly structured by Twilled weave fabrics, having weft yarns floating across the warp yarns of indigo-dyed cotton. These floating weft yarns help to impart better tactile comfort and thermal contact between the skin and the fabric surface and the influence of warp yarns that do not come in contact with the skin is minimal. Hence, weft yarns predominantly influence the denim fabric properties. For this purpose, blended denim fabrics with varying constructional parameters are being studded. The present study is an attempt to develop lightweight and comfortable denim products having variable weft-wise yarns. The study also suggested that the dyed weft yarns can be considered good candidate materials for newly developed denim products.

### Dyeing properties of warp indigo cotton yarn

**Scheme 1:**
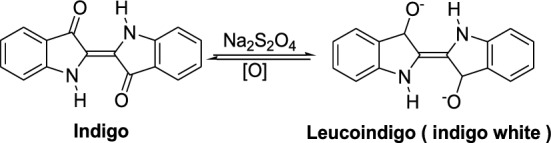
Conversion of the insoluble blue indigo dye to the water soluble leucoindigo.

**Figure 3:**
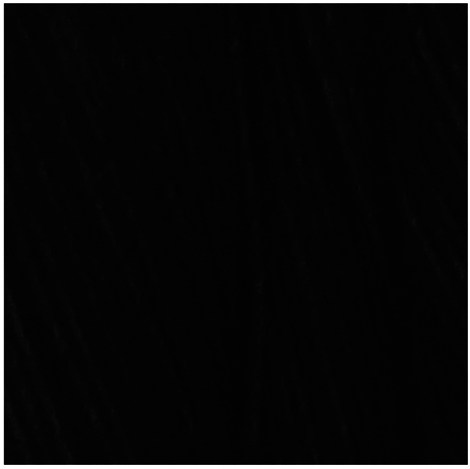
Photo image of the used warp cotton yarn indigo-dyed sample.

In the dyeing technique of warp cotton yarn, the insoluble indigo dye is reduced in an alkaline dyeing bath with sodium hydrosulfite (sodium dithionite) into the water-soluble leucoindigo form. When the reduced clear yellow leucoindigo solution comes into contact with air, it oxidizes back to the insoluble blue indigo compound, as shown in Scheme 1^32^. The color strength (K/S) and color coordinates data of the warp indigo-dyed cotton yarn sample, shown in Fig. 3, are given in Table 4.

**Table 4:** Color strength and colorimetric data of indigo-dyed cotton yarn.

K/S λ_max_ 615 nm	L*	a*	b*	C*	*h*
23.50	18.71	0.63	− 10.80	10.82	273.36

Where, L* represents lightness or darkness of the dyed sample, a* denotes the red/green value, b* the yellow/blue value, C* specifies chroma or saturation of the color and h° denotes hue angle.

### Dyeing properties of weft-wise silk yarns

#### Indigo Carmine dyeing

**Figure 4:**
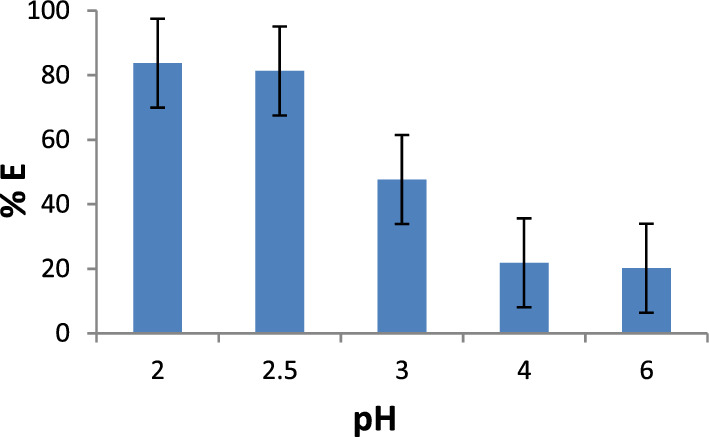
Dye exhaustion of Indigo Carmine on silk at different dyeing pH range.

**Figure 5:**
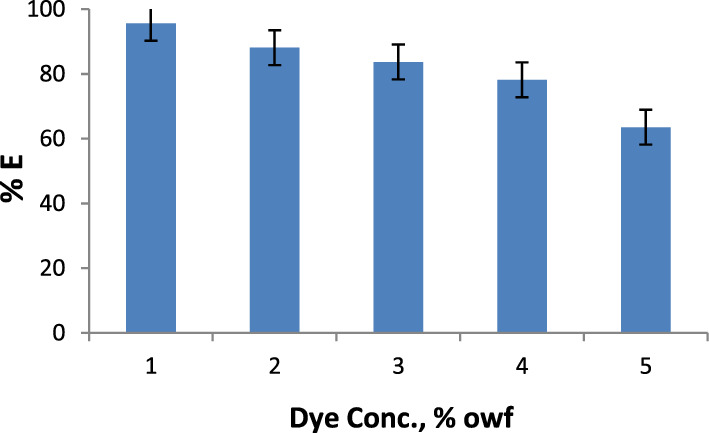
Dye exhaustion of Indigo Carmine on silk at different dye concentrations.

To ascertain the dyeing shades evaluation of the anionic Indigo Carmine on silk yarns, the dyeing process was conducted at various pH and dye concentrations as presented in Figs. 4, 5, and Tables 5, 6. Evaluation of the dyed silk samples indicates that the dye exhibited maximum exhaustion with relatively high exhaustion and color strength values at the acidic pH range of 2–2.5**.**

**Table 5:** Color data of Indigo Carmine dyed silk at different dyeing pH range.

pH	K/S λ_max_ 615 nm	L*	a*	b*	C*	*h*
2	13.53	37.38	− 9.65	− 26.95	28.63	250.30
2.5	12.94	38.04	− 11.90	− 26.63	29.16	245.92
3	11.35	41.53	− 13.80	− 25.82	29.28	241.87
4	3.15	57.29	− 16.49	− 17.12	23.77	226.08
6	1.72	64.56	− 15.62	− 12.55	20.04	218.79

**Table 6:** Color data of Indigo Carmine dyed silk at different dye concentrations.

Dye Conc. % owf	K/S λ_max_ 615 nm	L*	a*	b*	C*	*h*
1	5.87	49.27	− 14.75	− 23.43	27.69	237.80
2	12.70	39.69	− 12.65	− 26.59	29.44	244.55
3	13.53	37.38	− 9.65	− 26.95	28.63	250.30
4	15.58	34.71	− 9.10	− 27.04	28.53	251.40
5	19.22	31.97	− 8.00	− 26.55	27.72	253.24

With two sulphonic groups within the low molecular weight structure, the Indigo Carmine dye behaves like a levelling acid dye type and its dye molecules are not firmly bound to the fiber sites, reflecting on its poor to moderate washing fastness^39^. The dyed samples were subjected to cationic after-treatment process using the commercially available benzalkonium chloride quaternary ammonium compound to avoid such problem. However, the after-treated Indigo Carmine dyed silk samples showed significant color shifts with unsatisfactory ratings of color difference ΔE 14–16 related to the untreated Indigo Carmine-dyed sample, as shown in Table 7. Accordingly, the silk yarn dyed with Indigo Carmine, to some extent, is a fair match with the unique indigo warp-dyed cotton color that is a crucial industrial point.

**Table 7:** Influence of cationization after-treatment on the color parameters of the Indigo Carmine dyed silk yarn.

Dye Conc. % owf	Silk Dyed Sample Treatment	K/S λ_max_ (nm) 615/560 (Before/After)	L*	a*	b*	C*	*h*	ΔE
1	Before	5.87	49.27	− 14.75	− 23.43	27.69	237.80	16.31
	After	3.22	60.39	− 4.62	− 16.60	17.23	254.45	
2	Before	12.70	39.69	− 12.65	− 26.59	29.44	244.55	16.69
	After	5.82	51.16	− 0.13	− 22.58	22.58	269.66	
3	Before	13.53	37.38	− 9.65	− 26.95	28.63	250.30	16.13
	After	6.67	47.48	1.37	− 23.88	23.92	273.27	
4	Before	15.58	34.71	− 9.10	− 27.04	28.53	251.40	14.39
	After	7.47	44.40	2.95	− 25.60	25.77	276.58	
5	Before	19.22	31.97	− 8.00	− 26.55	27.72	253.24	15.24
	After	9.22	42.75	2.74	− 25.46	25.60	276.15	

#### Dyeing with non-indigo dyes

An attempt to dye silk yarn with matching indigo colors was studied using models of commercial blue reactive and acid dyes to avoid the un-matched color with Indigo Carmine dyeing. A simple reactive dyeing using the commercially available dye **RB 5** was performed on silk yarn under neutral pH. The dye exhibited a good dye uptake on silk, and its color data are listed in Table 8. The neutral dyeing conditions can further assist the dye-fiber interactions between the amino groups in silk and the bifunctional bis (VS) group, which may result primarily from the β-elimination of the bis-SES dye precursor^40,41^. The results also indicated that the color strength and color coordinate CIE L*, a*, and b* values of the neutral dyed silk samples are nearly at the same chromaticity zone with a little shade deviation in a* values, to those of the conventionally indigo-dyed warp cotton yarns, particularly at a high dye concentration of 4% (owf). On the other hand, a typical commercially available navy blue metal-complex acid dye **AB 193**, containing one sulfonic group (1S), was selected for its high performance to light fastness properties required in silk fiber dyeing. Having established that the optimum application pH of a typical acid dye on silk at a slightly acidic dyeing bath with a range of pH at 5–5.5, the color strength and color coordinates values of this dye seem to be matched to those of the conventional indigo-dyed warp cotton sample, as shown in Table 8.

**Table 8:** Color data of silk dyed with **RB 5** and **AB 193** at 2 and 4% owf depth of shades relative to the warp indigo-dyed cotton yarn.

Dye	Dye Conc. % owf	K/S	L*	a*	b*	C*	*h*
Indigo	2	23.50	18.71	0.63	− 10.80	10.82	273.36
**RB 5** λmax. 595 nm	2	17.42	24.89	− 4.23	− 12.46	13.16	251.23
	4	21.43	20.56	− 1.30	− 10.38	10.62	257.72
**AB 193** λ_max._575 nm	2	26.06	17.07	4.28	− 10.15	11.01	292.88
	4	33.76	13.18	2.08	− 4.16	4.65	296.59

#### Denim fabrics properties

The results listed in Table 9 show that the fabric weight of the control blend fabric followed the order: **DF3** > **DF2** > **DF1**. Sample **DF1**, which contains silk yarn in the weft direction is the lowest weight of the used fabrics having the same Twill pattern 3/1 Z. The use of silk yarn in the weft direction achieved good air permeability, and the fabric with satin weave structure **DF3** showed better performance than the Twill-based fabrics **DF1**, **DF2**. Importantly, the blend denim fabric **DF3** with satin structure gave better results in terms of the tearing strength and fabric air permeability compared to the other silk based samples. However, higher silk setting content in the weft direction exhibits not only better results for tearing strength but also the structure became less bulky and smoother with low fabric density and fabric stiffness compared to the samples **DF1** and **DF2**.

**Table 9:** Properties of control blend denim fabrics.

Control sample	Fabric weight g/m^2^	Thickness mm	Stetting (Warp/Weft) (cm^-1^)	Tearing strength (Warp/Weft) gf	Fabric density g/cm^3^	Fabric Stiffness (Warp/Weft) mg	Air permeability Cm^3^/cm^2^.sec
**DF1**	187	0.43	30/40	8650/8450	0.435	2590/1290	20.1
**DF2**	195	0.43	30/40	9050/8800	0.453	2714/756	19.6
**DF3**	197	0.45	30/40	9300/9500	0.419	2670/197	24.4

The effect of using weft setting of undyed silk yarn related to the different weave patterns of control blend denim fabric are presented in Table 10.

**Table 10:** Control blend denim fabrics of warp dyed cotton yarn and un-dyed silk yarn.

Control sample code	Weave pattern	View	
		Front	Back
**DF1**	Twill 3/1 Z		
**DF2**	Twill 3/2 Z		
**DF3**	Satin 5_3_		

The viability of using silk yarn in the weft setting of different weaved patterns has been proposed and successfully introduced as weft-wise dyed silk yarn in two sets of blend fabrics **A** and **B**. Each set of the blend fabrics developed in Twill 3/1, 3/2 and Satin 5_3_ patterns. Set **A** fabrics included **DF1A**, **DF2A** and **DF3A**, in which silk yarns were subjected to dyeing with reactive dye (**RB 5**), at 4% owf shade. While Set **B** of the blend fabrics **DF1B**, **DF2B** and **DF3B** included silk yarns were subjected to dyeing with acid dye (**AB 193**), at 2% owf shade. The production parameters concerning the weaving process of each set of samples were identical to those of the control fabrics **DF1**, **DF2** and **DF3**, respectively. The front/back views of these fabric structures are showed in Table 11.

**Table 11:** Specifications of dyed silk weft setting in blend denim fabrics.

Dye set	Sample code	View	
		Front	Back
**A**	**DF1A**		
	**DF2A**		
	**DF3A**		
**B**	**DF1B**		
	**DF2B**		
	**DF3B**		

Concerning the color data listed in Table 12, the blending of undyed weft silk yarn used in the structure of fabric was analyzed. The color strength of the satin-based weaved fabric exhibited higher values than the twilled fabrics. The color effects of the control fabrics depend on the fabric structure and the composition of indigo-dyed cotton yarn. Here, the high the indigo-dyed cotton yarn into the surface of the satin sample is mainly responsible for the highest color strength value. Moreover, **DF1** gives better results than **DF2** due to the presence of the higher content of dyed cotton yarn on fabric surface of the twilled 3/1 Z fabric compared with 3/2 Z.

**Table 12:** Color strength, colorimetric data and UPF values of silk blend denim fabrics.

Sample code	View	K/S λ_max_**	L*	a*	b*	C*	*h*	Δ E	UPF
**DF1**	Front	6.92	30.21	0.03	− 4.46	4.46	270.32	–	321.0
	Back	1.83	52.14	− 1.86	− 397	4.38	244.88	22.02	322.5
**DF2**	Front	4.19	39.85	− 1.09	− 4.27	4.41	255.65	–	715.8
	Back	2.47	47.82	− 1.58	− 3.69	4.01	246.83	8.00	486.7
**DF3**	Front	10.53	25.77	0.40	− 4.38	4.39	275.21	–	258.7
	Back	1.29	57.70	− 2.19	− 3.70	4.30	239.40	32.04	510.6
**DF1A**	Front	22.44	18.83	− 0.23	− 8.91	8.92	268.51	–	934.7
	Back	15.95	26.49	− 4.99	− 13.03	13.95	249.05	9.92	1015.6
**DF2A**	Front	22.05	21.11	− 2.24	− 10.85	11.08	258.31	–	9810.8
	Back	17.69	24.61	− 3.98	− 12.42	13.04	252.23	4.21	22,241.3
**DF3A**	Front	26.85	16.78	1.00	− 8.03	8.10	277.10	–	1943.3
	Back	20.07	23.97	− 4.37	− 13.14	13.85	251.62	10.32	1430.0
**DF1B**	Front	26.92	16.69	1.34	− 8.46	8.56	279.02	–	1060.6
	Back	31.66	15.14	1.84	− 6.39	6.68	286.86	2.66	1354.0
**DF2B**	Front	26.52	16.60	1.42	− 8.10	8.22	279.97	–	10,531.1
	Back	29.48	15.38	1.66	− 7.30	7.48	282.79	1.48	27,149.4
**DF3B**	Front	30.21	15.39	1.66	− 8.16	8.33	281.53	–	10,578.7
	Back	30.16	14.57	1.91	− 5.54	5.86	289.02	2.76	14,589.3

** λ_max_ control samples: 625 nm, Set **A** : 595 nm and λ_max_ Set **B**: 575 nm.

Further investigation of the produced fabrics made of dyed silk yarns, the color effects of the developed blend denim products of sets **A** and **B** were evaluated. From which, the dyeing performance of the blend denim fabrics of set **B** that were dyed with **AB 193** (**DF1B, DF2B** and** DF3B**), showed better results of the color strength and colorimetric data with minimal front/back color difference values than the fabrics produced in set **A**, as listed in Table 12.

The UPF values of the blend denim fabrics of the dyed weft-wise silk yarns samples showed significant improvement over those of the undyed silk, as given in Table 12. It is noticed that the fabric density which follows the order **DF2** > **DF1** > **DF3** and its corresponding air permeability order **DF2** < **DF1** < **DF3** in the control blend fabrics, as given in Table 9, the higher fabric density and lower air permeability of the control fabric **DF2** and its dyed silk based blend fabrics **DF2A** and **DF2B** showed high UPF values. Additionally, acid-dyed silk based blend fabrics **DF1B**, **DF2B** and **DF3B** showed significantly higher UPF values than those of the reactive-dyed silk based blend fabrics **DF1A**, **DF2A** and **DF3A**. Thus, it can be inferred that the dye molecules is the key responsible for the screen effect against UV exposure with an excellent protection level when using undyed weft yarns in the construction of denim fabric.

#### Fastness properties

The fastness properties of all the developed blend denim fabrics are listed in Table 13. The acid- dyed silk samples of the denim fabrics **DF1B**, **DF2B** and **DF3B** displayed excellent fastness to light fastness. However, the colorfastness to light in the case of reactive-dyed samples was slightly lower, as the **RB 5** dye does not have as good stability against light as the Cr-metalized chromophoric system of the **AB 193** acid dye. The two sets-based blend denim fabrics gave very good to excellent colorfastness ratings to wash and perspiration with almost the same grades on both front and back sides to the fastness properties of the typical front control samples.

**Table 13:** Fastness properties of the blend denim fabrics.

Sample code	View	Washing fastness			Perspiration fastness						Light
					Acidic			Alkaline			
		*CC*	*SC*	*SW*	*CC*	*SC*	*SW*	*CC*	*SC*	*SW*	
**DF1**	front	4–5	4–5	4–5	4–5	5	4–5	4–5	5	4–5	6–7
**DF2**		4–5	4–5	4–5	4–5	5	4–5	4–5	5	4–5	6–7
**DF3**		4–5	4–5	4–5	4–5	5	4–5	4–5	5	4–5	6–7
**DF1A**	Front	4–5	5	4–5	4–5	4–5	4–5	4–5	4–5	5	5
	Back	4–5	5	4–5	4–5	4–5	4–5	4–5	4–5	4–5	5
**DF2A**	Front	4–5	5	4–5	4–5	4–5	4–5	4–5	4–5	4–5	5
	Back	4–5	5	4–5	4–5	4–5	4–5	4–5	4–5	4–5	5
**DF3A**	Front	4–5	5	4–5	4–5	4–5	4–5	4–5	4–5	4–5	5–6
	Back	4–5	5	4–5	4–5	4–5	4–5	4–5	4–5	4–5	5
**DF1B**	Front	4–5	5	5	4–5	5	5	4–5	5	5	6–7
	Back	4–5	5	5	4–5	5	5	4–5	5	5	6–7
**DF2B**	Front	4–5	5	5	4–5	5	5	4–5	5	5	6–7
	Back	4–5	5	5	4–5	5	5	4–5	5	5	6–7
**DF3B**	Front	4–5	5	5	4–5	5	5	4–5	5	5	6–7
	Back	4–5	5	5	4–5	5	5	4–5	5	5	6–7

*CC = Change of color; *SC* = Staining on cotton; *SW* = Staining on wool.

## Conclusion

The present study attempted to develop lightweight blended denim products having weft-wise silk yarn produced in 3/1Z, 3/2Z Twill and Satin 5_3_ weave structures. Control blend denim fabrics are manufactured by weaving indigo-dyed cotton warp yarns with weft yarns of undyed silk fibers. The weaving of silk yarn in the weft direction achieved good air permeability and better performance with Satin fabric than Twill blend fabrics. The fabric sample of higher silk setting in the weft direction exhibited better results for tearing strength, in addition to the fabric structure becoming less bulky and smoother with low density and stiffness. The study also concluded that the dyed weft silk yarns claimed plausible candidate materials for newly developed denim products. To ascertain the dyeing shades evaluation of the anionic Indigo Carmine on silk, the dye exhibited maximum exhaustion values with relatively high build-up on silk at the highly acidic pH range 2–2.5, which behaves like a levelling acid dye type but its dye molecules are not firmly bound to the fiber sites, imparting poor to moderate washing fastness. The dyed silk samples were subjected to cationic after-treatment and showed high color differences of Δ E 14–16. Accordingly, to match the colors of the indigo dye, the silk yarn was dyed with selected commercial non-indigo reactive and acid dyes. Two sets of blend denim fabrics were fabricated in 3/1 and 3/2Z Twill and Satin weave structures. One set of fabrics is reactive-dyed silk yarns, while the second is acid-dyed silk. The dyeing performance of the blend denim fabrics made of acid-dyed silk yarn showed better results of color strength and colorimetric data with minimal front/back color difference values and excellent light fastness. The UPF of the blend fabrics of dyed silk yarns showed significant improvement in the ultraviolet protection factors. The two sets-based blend denim fabrics were categorized as very good to excellent colorfastness to washing and perspiration with almost the same grades as the control samples. Overall, dyed weft-wise silk yarns blended with the warp-dyed indigo cotton yarn can impart acceptable and satisfied candidate materials for newly developed blend denim fabrics.

## Data Availability

All data generated or analyzed during this study are included in this article.
